# Overproduction of a thermo-stable halo-alkaline protease on agro-waste-based optimized medium through alternate combinatorial random mutagenesis of *Stenotrophomonas acidaminiphila*

**DOI:** 10.1016/j.btre.2022.e00746

**Published:** 2022-06-05

**Authors:** Atim Asitok, Maurice Ekpenyong, Iquo Takon, Sylvester Antai, Nkpa Ogarekpe, Richard Antigha, Philomena Edet, Ubong Ben, Anthony Akpan, Agnes Antai, Joseph Essien

**Affiliations:** a Environmental Microbiology and Biotechnology Unit, Department of Microbiology, Faculty of Biological Sciences, University of Calabar, Nigeria; b University of Calabar Collection of Microorganisms (UCCM), Department of Microbiology, University of Calabar, Nigeria; c Industrial Microbiology and Biotechnology Unit, Department of Microbiology, Faculty of Biological Sciences, University of Calabar, Nigeria; d Environmental Engineering Unit, Department of Civil Engineering, Faculty of Engineering, Cross River University of Technology, Nigeria; e Department of Physics, Faculty of Physical Sciences, University of Calabar, Nigeria; f Department of Economics, Faculty of Social Sciences, University of Calabar, Nigeria; g Environmental Microbiology and Biotechnology Unit, Department of Microbiology, Faculty of Sciences, University of Uyo, Nigeria

**Keywords:** Mutagenesis, Cassava waste-stream, Alkaline protease, Neural network optimization, Aqueous two-phase system, Detergency applications

## Abstract

A strain of *Stenotrophomonas acidaminiphila*, isolated from fermenting bean-processing wastewater, produced alkaline protease in pretreated cassava waste-stream, but with low yield. Strain improvement by alternate combinatorial random mutagenesis and bioprocess optimization using comparative statistical and neural network methods enhanced yield by 17.8-fold in mutant kGy-04-UV-25. Kinetics of production by selected mutant modeled by logistic and modified Gompertz functions revealed higher specific growth rate in mutant than in the parent strain, likewise volumetric and specific productivities. Purification by PEG/Na^+^ citrate aqueous two-phase system recovered 73.87% yield and 52.55-fold of protease. Its activity was stable at 5–35% NaCl, 45–75°C, and was significantly enhanced by 1–15 mM sodium dodecyl sulfate (SDS). The protease was inhibited by low concentrations of phenyl-methyl-sulfonyl fluoride but was activated by 1–5 mM Mn^2+^ suggesting a manganese-dependent serine‑protease. The 45.7 kDa thermo-halo-stable alkaline protease demonstrated keratinolytic and blood-stain removal potentials showing prospects in textile and detergent industries, respectively.

## Highlights

• Alternate combinatorial random mutagenesis selected a protease high-yielding mutant.
• Medium optimization led to 25.55-fold raise in specific protease yield in bioreactor.
• 20% PEG-1500/Na+ 15% citrate recovered 74% activity yield with 52.55 purity.
• Activity was retained at elevated physicochemical levels but inhibited by PMSF.
• Keratinolytic and blood-stain removal activities confer industrial potential on protease.

## 1 Introduction

Proteases, or more specifically peptidyl-peptide hydrolases (EC 3.4.21–24 and 99), constitute 60–65% of global enzyme market [1, 2] on account of their tremendous applications in detergent, tannery, food, pharmaceutical, medical, leather industries and environmental bioremediation [3, 4]. Biochemically speaking, they catalyze the hydrolytic cleavage of peptide bonds in proteins partially generating oligopeptides of various sizes and more completely amino acids. Although plant and animal proteases exist, microbial sources are commercially preferred owing to ease of production, recovery and amenability to improved production especially [5].

Microbial proteases are broadly classified, in terms of pH discrepancy, as acidic, neutral and alkaline. While acid proteases, produced mainly by filamentous fungi, are less frequently reported [6], alkaline proteases, produced mostly by bacteria, dominate literature because of their characteristics are most desired in various industries. The quest, therefore, for alkaline proteases for industrial operations have been intense for more than five decades and continues unabated.

The bacterial genus, *Bacillus* has been at the forefront of laboratory and commercial production of proteases. Species like *B. licheniformis, B. cereus, B. subtilis, B. safensis*, etc. have carved commercial niches for themselves [7]. However, baseline yields have been low arising from tight control/regulation of the biosynthetic pathway of the enzyme and poor understanding of the optimum nutritional and environmental requirements of the producing organism. Accordingly, organisms with loose metabolic control, otherwise called over-expressing strains, especially from non-*Bacillus* group like *Stenotrophomonas* [8, 9] and *Idiomarina* [10] are vigorously investigated. Alternatively, genetic manipulations have been employed to loosen tight metabolic control of some promising producer strains to improve yield. Reported successful strain improvement methods include random physical or chemical mutagenesis, site-directed mutagenesis, protoplast fusion as well as recombinant DNA technology [11, 12].

Design of experiment, involving response surface methodology (RSM) and artificial neural network (ANN) methods, has been employed to address the nutritional problems of protease-producing microorganisms, where meeting of significant requirements of microorganisms is prioritized. A major concern during medium development has been cost but exploitation of renewable, especially agro-industrial wastes, as carbon and nitrogen substrates in fermentation media has been most helpful [13, 14].

The major objectives of this study were to improve protease yield by the non-*Bacillus* strain; *Stenotrophomonas acidaminiphila* UCCM 00,065, using the synergy of alternate combinatorial random mutagenesis and medium optimization, and to demonstrate its applications in detergent and leather industries. Major experiments conducted include (i) strain improvement (ii) Comparative optimization by RSM and ANN (iii) bioreactor fermentation kinetics study (iv) aqueous two-phase system purification and stability evaluations of protease and (v) evaluation of potential industrial applications.

## 2 Materials and methods

### 2.1 Strain improvement of producing bacterium by alternating combinatorial random mutagenesis

*Stenotrophomonas acidaminiphila* strain UCCM 00,065 with 99.4% sequence similarity with *Stenotrophomonas acidaminiphila* strain AMX19 (GenBank No.: AF273080), was retrieved from the University of Calabar Collection of Microorganisms (UCCM) as a -80°C glycerol-frozen stock (20% [wv^−1^] glycerol/2% [wv^−1^] peptone) and reactivated according to collection's guidelines (www.wfcc.info/ccinfo/collection/by_id/652).

Two physical mutagens, namely ultraviolet and gamma rays were employed in alternate combinatorial fashion to improve protease production by the bacterium. For UV-irradiation, bacterial populations of 10^8^ cellsmL^−1^ were exposed at 15, 25 and 35 cm distance from UV-lamp (254 nm, 15 W, 2537A^0^) for 30 min. Gamma irradiation was conducted at the Multipurpose Gamma Irradiation Facility (GIF) of the Nuclear Technology Center (NTC), Sheda Science and Technology Complex, Abuja, Nigeria. Cobalt 60 (^60^Co) from Indian Gamma Chamber 4000A was used at variable doses ranging from 0 to 3 kGy with 0.2 kGy increments at an exposure distance of 100 cm for 30 min. In both cases, irradiated cells were serially diluted (10-fold) in normal saline (0.90% NaCl) and plated on trypticase soy agar (TSA) by the spread plate technique and incubated at 30°C for 48 h in the dark to obtain viable cells. All viable cells were screened on freshly prepared skimmed-milk-minimal agar medium containing 1% skimmed milk (Merck); 0.02% MgSO_4_·7H_2_O; 0.01% FeSO_4_·7H_2_O; 0.1% K_2_HPO_4_; 0.1% KH_2_PO_4_; 3% NaCl; 1.5% (wv^−1^) agar-agar. Plates were examined for zones of clearance within 24 h of incubation at room temperature (28 ± 2°C) and cells with clear zones significantly larger than the 3.0 cm of parent strain were considered improved and selected for confirmation in submerged culture.

In the submerged fermentation, minimal medium was dispensed as 20 mL into 100 mL Erlenmeyer flasks in triplicates. Flasks were sterilized by autoclaving and supplemented with filter-sterilized skimmed milk after cooling and subsequently inoculated with 3% (vv^−1^) 18 h-old Luria-Bertani (LB) broth of all improved mutants. The preparation was incubated at room temperature (28 ± 2°C) on shaker incubator (Gallenkamp, UK) agitating at 150 rpm for 36 h. Protease was harvested by centrifugation at 10,621 x *g* at 4°C for 15 min, followed by filtration (0.22 µm, Millipore). Total protein and protease activity were determined from the sterile filtrate according to Bradford method [15] and Asitok et al. [16]. All improved strains were exposed a second time, but in alternate fashion, to either of the two mutagens: UV-selected mutants to gamma irradiation and gamma-improved mutants to UV irradiation. Screening was as described earlier and improved mutants were maintained on agar slopes in the refrigerator for further studies.

### 2.2 Treatment, composition and preparation of cassava processing effluent

Cassava processing effluent was collected from a local processing industry in Calabar, Nigeria. The CPE was first boiled, then allowed to cool to 5°C. The cooled waste-stream was centrifuged at 1699 x *g* for 20 min to facilitate removal of suspended solids and elimination of cyanogenic compounds especially hydrogen cyanide. The effluent was then sterilized by autoclaving at 121°C for 15 min to eliminate persistent undesirable microorganisms. Upon cooling, the sterile gelatinized effluent was treated with a bacterio-fungal cocktail in sequence: first with 3% (vv^−1^) of an overnight (18 h) Luria-Bertani (LB) broth culture (10^8^ cfumL^−1^) of *Bacillus licheniformis* strain UCCM 00,096 and incubated for 48 h at 35°C for controlled liquefaction of starch. The preparation was sterilized and upon cooling, inoculated with 3% (vv^−1^) spore suspension (10^8^ sfumL^−1^) of *Aspergillus niger* strain UCCM 00,114 and incubated at 30°C for 72 h for saccharification of the liquefied starch. The resulting treated CPE was concentrated by evaporation at 45°C. Total reducing sugar was determined by the di-nitro-salicylic (DNS) acid method [17] for all three stages of pre-treatment.

### 2.3 Design of experiment (DoE) and optimization by response surface methodology and artificial neural network

The two-level factorial design (2-LFD) was employed to screen significant variables for inclusion in fermentation medium. Thereafter, a path of steepest ascent (PSA) experimentation was conducted using coefficients of significant factors obtained from the first-order model of 2-LFD to move variable levels through calculated step change sizes towards the optimum. The first-order model equation is given as;

(1) $$y=b_{0}+\sum b_{i}x_{i}+\sum b_{\mathrm{ij}}\sum x_{i}x_{j}+\varepsilon$$

where *y* is total protein, *b*_0_, a constant term, *b*_i_, coefficient of linear term and *b*_ij_ coefficient of interaction term, *x*_i_, *x*_j_ for investigated variables and *ɛ* the error term.

A central composite rotatable design (CCRD) matrix of a surface methodology (RSM) was adopted to locate the optimum levels of factors within the narrow ranges specified by the PSA. Fifty experimental runs involving 5 input variables and 1 response variable were set up using full-factorial design in Design Expert 12 (Stat Ease, Minnesota, USA). The experiments were divided into 6 replicated runs at center points to test the fit of model, 10 runs at axial points and 34 experimental runs at factorial points. Total protein was related to input variables by the second-order polynomial function below:

(2) $$y=\beta_{0}+\sum_{i=1}^{k}\beta_{i}x_{i}+\sum_{i=1}^{k}\beta_{ii}x_{i}^{2}+\beta_{ij}x_{i}x_{j}+\varepsilon$$

where y is protease activity, *β_0_, β_i_, β_ii_* and *β_ij_* are coefficients of the constant, linear (*x*_i_), quadratic (*x*_i_^2^) and interaction terms (*x*_i_*x*_j_) of *k* factors respectively, with ɛ as error term of computation of total protein (protease). The statistical significance of the relationship was tested using the ANOVA model at 95% confidence level.

Data obtained from the 50 experimental runs from CCRD were comparatively modeled by response surface methodology (RSM) and artificial neural network (ANN) to predict the optimum levels of the independent variables that would yield maximum protease. Artificial neural network is a computer-based simulation of the biological coordination of the human brain to solve complex non-linear problems such as bioprocess optimization. Its success lies in the choice of algorithm and careful selection of number of interconnecting neurons. In this study, Levenberg-Marquardt back-propagation algorithm of the neural fitting application in Matlab 2014a software (Mathworks Inc. USA) was chosen to select data, create, train, validate and test network. The network was a two-layer feed-forward neural network with sigmoid hidden neurons and linear output neurons with inbuilt ability to fit a multi-dimensional mapping problem.

The 50 samples were presented to the algorithm and, by default, were randomly divided into 70% (34) for training by adjusting the weights and biases feeding from the 5 input variables in the input layer, 15% (8) for validation and 15% (8) for testing the performance of the network during and after training. The optimal performance of the network was scored on the basis of low root mean squared error (rmse) and high coefficient of determination, *r*^2^. All data for training, validation and testing of the network was normalized between -1 and +1 using the equation below.

(3) $$y=\frac{2x_{i}-\left(x_{max}+x_{min}\right)}{x_{max}-x_{min}}$$

where y is the normalized value of *x*_i_ and *x*_max_ and *x*_min_ are the respective maximum and minimum values of *x*_i_.

The predictive capabilities of CCRD-RSM and CCRD-ANN models were compared using model performance metrics including coefficient of determination, *r*^2^, mean squared error (MSE), root mean squared error (RMSE), mean absolute error (MAE), average absolute deviation (AAD), Average relative error (ARE) and Pearson's Chi squared error (χ2). The best modeled conditions for maximum yield of total protein were optimized by the response optimizer in Design Expert 12 using the desirability function and variable importance.

### 2.4 Confirmation experiment

The optimum conditions suggested by optimization were employed to set up fresh triplicate fermentations in 1-L Erlenmeyer flask for ratification. Total protein was determined as previously described and a difference of less than 5% in protease concentration between optimization and confirmation experiment ratified the model as suitable for future predictions of protease production by the mutant.

### 2.5 Bioreactor production of protease by Stenotrophomonas acidaminiphila mutant in optimized medium

Batch-mode fermentative production of protease by most improved mutant of *Stenotrophomonas acidaminiphila* under optimized medium conditions was conducted in a 5-L bench-scale bioreactor (BioStat, Sartorius) with a working volume of 3.5 L. Fermentation medium had the same composition as that used in the confirmation experiment. Temperature was left at 31°C, pH 7.0, agitation 150 rpm and dissolved oxygen 50%. Filter-sterilized air was allowed to flow into the headspace of the vessel at a rate of 1 Lmin^−1^ regulated by a mass flow controller. The bioreactor was equipped with a single in-place sterilization mechanism. The vessel was allowed to cool near to 31°C after sterilization before inoculation, through culture inlet, with 3% (vv^−1^) overnight LB broth culture of the mutant bacterium. The bioreactor was operated for 0, 4, 8, 12, 16, 20, 24, 28, 32 and 36 h.

### 2.6 Determination of fermentation response variables

Fermentation broth from each triplicate batch was centrifuged at 10,621 x *g* at 4°C for 15 min and determinations of total protein, protease activity, biomass concentration and amount of substrate consumed were made from appropriate fractions.

Total protein was determined from sterile supernatant by the Bradford method using bovine serum albumin as standard protein and coomasie brilliant blue G-250 dye as protein reagent [15]. Triplicate determinations were obtained using the regression equation of the standard curve: $TP=(OD_{595}-0.7533)/0.0109$, $R^{2}=0.9934$

Protease activity was determined from sterile supernatant by the method described in Asitok et al. [16] using azocasein as substrate.

Biomass was quantified by the dry cell weight (DCW) technique [18] from the pellet obtained from centrifugation at 10,621 x *g* for 5 min. The relationship between dry cell weight (DCW) and optical density at 600 nm was given by the expression $DCW=(OD_{600}\times \;0.633)+0.031$, $r^{2}=0.9984$.

Amount of substrate consumed was determined by the DNS assay [17] with glucose (Sigma Aldrich, USA) as standard using the expression $OD_{540}=0.023x+0.0282$, $r^{2}=0.9991$.

Triplicate time-related response data were subjected to descriptive statistics to obtain mean parameters +/- standard error. Means were compared by one-way ANOVA and significant means separated by Tukey's HSD post-hoc multiple comparisons test using 95% confidence interval.

### 2.7 Kinetic model-fitting of experimental data and model-performance evaluation

Mean data for biomass concentration was fitted to the logistic model [18] while data for total protein and substrate consumption were fitted to modified Gompertz model as described in Ekpenyong et al. [19].

Kinetic parameters were obtained in triplicates and results reported as mean +/- standard error. Model performances were evaluated using RMSE, MAE and adjusted coefficient of determination, *r*^2^. Significance of models was tested at 95% confidence level.

### 2.8 Purification of alkaline protease by aqueous two-phase system

Polyethylene glycol-sodium citrate (PEG-Na^+^Citrate) system was employed for aqueous two-phase system purification of the enzyme. Five different sizes (molecular weights) of the PEG including 1500, 3000, 4500, 6000 and 7500 were used. The component phase systems were constituted by%ww^−1^ in a 15 mL graduated centrifuge tube. Total mixture of polymer, water, sodium citrate and 2% crude enzyme in the tube was 100%. Separation was conducted at pH 10.5 of sodium citrate and holding temperature of 65°C. The mixture was centrifuged at 956 x *g* for 20 min to facilitate phase separation and tube held at 20°C for 24 h for equilibration [20]. Determinations of volume ratio, partition coefficient, yield and enzyme purification were as described in Sarangi et al. [21].

### 2.9 Determination of molecular weight of alkaline protease by SDS-PAGE

The molecular weight of the aqueous two-phase purified protease from *Stenotrophomonas acidaminiphila* strain kGy-04-UV-25 was re-determined according to the method of Laemmli [22].

### 2.10 Evaluation of the effects of temperature, pH, NaCl, denaturants, divalent cations, organic solvents and inhibitors on protease activity and stability

Stability of protease to temperature and pH were evaluated after determining optimum temperature and pH from ranges 35–95°C and 4.5–12.5 respectively. For thermal stability, the enzyme was first exposed in 50 mM Tris–HCl buffer (pH 7.5) to temperatures of 65–85°C for 0–75 min and then placed back on ice to refold for 15 min before measuring residual activity. Stability to different pH levels from 6.5 to 11.5 at 4°C for 0–75 min was evaluated as in Iboyo et al. [23]. Sodium bicarbonate-sodium hydroxide and potassium chloride-sodium hydroxide buffers were employed for pH 11.5–12.5 [24]. Stability to NaCl (0–40%) for 15–75 min, was evaluated by pre-incubation of crude protease under the specified conditions of NaCl before determining relative activity.

Stability to denaturants was evaluated by pre-incubating the enzyme with sodium dodecyl sulfate (SDS), triton X-100, Tween 80 and Tween 20 each at 1, 5 and 10 mM for 1 h before repeating protease assay and determining relative activity. Effect of divalent cations was investigated by prior exposure of the crude enzyme to 1 mM concentrations of Ca^2+^, Mg^2+^, Ba^2+^, Zn^2+^, Ni^2+^, Mn^2+^, Fe^2+^, Co^2+^, Cu^2+^ and Pb^2+^ for 15 min before repeating protease assay. Stability of purified protease to organic solvents was tested against 50% (vv^−1^) methanol, dimethylsulfoxide, acetone, acetonitrile, chloroform, hexane, cyclohexane and toluene at 65°C and 150 rpm agitation for 24 h. Thereafter, residual activities were determined using protease without solvent as control. Finally, purified protease was exposed to 5 mM of phenyl-methyl-sulfonyl fluoride (PMSF), ethylene diamine tetraacetic acid (EDTA), dithio-bis-nitrobenzoic acid (DTNB), *p*‑chloro-mercuribenzoate (*p*CMB) and dithiothreitol (DTT), and 5% (v/v) β-mercaptoethanol (β-MEOH). Inhibitor poisoning was held for 15 min before determining residual activity of the protease.

### 2.11 Evaluation of potential applications of protease as detergent additive

Evaluation of detergent additive potential of the protease was performed using blood stain removal protocol of Mothe and Sultanpuram [25]. Briefly, white cotton cloth was cut into 4 pieces (10 × 10 cm) and stained with 10 drops of fresh sheep's blood. Cloth pieces were dried in an oven at 95°C for 5 min and subsequently washed as follows:

i 100 mL distilled water + a piece of blood stained cloth + 1 mL of commercial detergent (5 mg/mL) + 1 mL of purified protease
ii 100 mL distilled water + a piece of blood stained cloth + 1 mL of commercial detergent (positive control)
iii 100 mL distilled water + a piece of blood stained cloth + 1 mL purified protease
iv 100 mL distilled water + a piece of blood stained cloth (negative control)

All treatments were incubated at 50°C for 30 min but taken out at 5 min interval, rinsed in tap water, dried at 95°C and then examined visually.

### 2.12 Compatibility with commercial detergents

Enzyme stability was also tested against commercial laundry detergents like OMO (Colgate-Palmolive, France), ARIEL (Procter and Gamble, Switzerland) and SUNLIGHT (Sun Products, USA). Detergents were added (7 mgmL^−1^) to tap water and then pre-incubated with crude enzyme for 1 h at 55, 65 and 75°C before determining residual activity. Preparation without commercial detergent was used as control and enzyme activity scored as 100%.

### 2.13 Keratinase activity of crude protease

Analytical keratin was prepared according to the modified Wawrzkiewicz method described in Mazotto et al. [26] from delipidated feather. Keratinase activity was determined according to Grzywnowicz et al. [27]. The reaction mixture contained 1 mL of a 1-in-4 dilution of sterile crude enzyme and 1.5 mL of 0.67% (w/v) keratin suspended in 0.1 M phosphate buffer at pH 6.5. The reaction was incubated for 1 h at 65°C and stopped by adding 1 mL of 10% trichloroacetic acid (TCA). This was held at 4°C for 30 min. A control in which 1 mL of TCA was added before incubation was also set up. The preparation was centrifuged at 2010 x *g* for 10 min and absorbance read off a spectrophotometer at a wavelength of 280 nm. One unit of keratinase activity was defined as the amount of enzyme required to produce an absorbance increase of 0.01 under assay conditions.

## 3 Results and discussion

### 3.1 Strain improvement by alternate combinatorial random mutagenesis

Rational screening and genetic engineering may make superior contributions to strain improvement but random screening and selection remains a cost effective, short-term and reliably preferred option. Results of the alternate combinatorial random mutagenesis of *Stenotrophomonas acidaminiphila* strain UCCM 00,065 using ultraviolet and gamma irradiation are presented in Table 1. Only a few strains had improved protease activity by single mutagen exposure: three each from either exposure. Best mutants had 50% (kGy-04) and 40% (UV-35) improvement from parent strain. However, in a second round of random mutagenesis, completely different improved mutants emerged. Mutant kGy-04-UV-25 recorded 137.16% improvement in protease activity which was followed by 92.84% improvement by mutant kGy-08-UV-25. It was interesting to observe that significant improvement in protease activity occurred when cells were first exposed to gamma rays before following up with ultraviolet exposure. In an alternate combination where cells were first exposed to UV-light before following up with gamma-irradiation, improvements were lower (Table 1). Random mutagenesis has the tendency to create variants in terms of alteration of the overall protein secretion pathway and more specifically the protein expression cascade or a combination of both. Mutant kGy-04-UV-25 demonstrated highest total protein suggesting up-regulating mutations in the extracellular protein secretion pathway genes [28]. The mutant also demonstrated highest protease activity suggesting accumulation of up-regulating mutations that specifically activated transcription of protease structural genes into functional mRNA molecules. Mutant kGy-04-UV-25 was accordingly selected on the basis of superior improvement from 36,209.54 U (123.73 mg protein) in the parent strain to 85,873.38 U (284.93 mg protein); some 2.3-fold increase in protein yield and 2.37-fold improvement in activity units. Many authors have reported successful improvement of alkaline protease by UV [29] or gamma irradiation [30], especially in *Bacillus* species. However, there are only a few reports on combinatorial mutagenesis [31] but none on alternate combinations. Mutagenesis by gamma irradiation is reported to cause ruptures in DNA strands leading to oxidation of nitrogenous bases and formation of DNA-protein cross linkages. These changes may compromise cellular repair mechanisms thus stabilizing thymine dimerizations induced by follow-up UV-irradiation. Since the reversed mutagenesis in two of the mutants yielded lower protease, a requirement to prioritize the ordering of mutations in a combinatorial mutagenesis approach is strongly recommended for further investigation.

**Table 1:** Improvement of alkaline protease production from *Stenotrophomonas acidaminiphila* strain UCCM 00,065 by alternate combinatorial random mutagenesis.

S/N	Mutant code	Mean diameter of clear zone (cm) ± SD	Mean total protein (mg)± SD	Mean protease activity (U) ± SD	% improvement in protease activity
1	Parent	3.0 ± 0.36	123.73 ± 10.65	36,209.54 ± 439.24	–
2	UV-15	3.1 ± 0.18	132.11 ± 9.58	41,893.09 ± 443.19	15.70
3	UV-25	3.2 ± 0.11	139.27 ± 12.11	45,904.73 ± 463.28	26.78
4	UV-35	3.6 ± 0.15	179.38 ± 15.37	50,784.87 ± 518.37	40.25
5	kGy-02	3.7 ± 0.26	198.36 ± 16.42	54,086.17 ± 419.47	49.37
6	kGy-04	3.4 ± 0.33	159.64 ± 16.35	54,396.62 ± 362.46	50.23
7	kGy-08	3.3 ± 0.21	148.22 ± 12.36	49,889.05 ± 528.47	37.78
8	UV-15-kGy-04	3.3 ± 0.52	142.67 ± 11.89	49,829.29 ± 392.83	37.61
9	UV-25-kGy-04	3.7 ± 0.47	194.36 ± 17.62	58,824.17 ± 653.22	62.46
10	UV-25-kGy-08	3.7 ± 0.14	201.36 ± 19.17	62,291.72 ± 572.81	72.03
11	kGy-04-UV-25	4.2 ± 0.32	284.93 ± 21.89	85,873.38 ± 783.84	137.16
12	kGy-06-UV-25	3.8 ± 0.38	218.84 ± 16.28	66,039.55 ± 689.83	82.38
13	kGy-08-UV-25	3.9 ± 0.63	223.14 ± 15.15	69,825.59 ± 701.26	92.84

UV – Ultraviolet exposure at 15, 25 and 35 cm from irradiation source; kGy – Gamma rays of 0.4, 0.6 and 0.8 kg Grays from a fixed distance of 100 cm from irradiation source; SD – standard deviation from mean of triplicate determinations; U – enzyme activity unit.

### 3.2 CCRD-RSM-ANN modeling and optimization

The two-level factorial design (2-LFD) selected 5 factors namely cassava processing effluent (CPE), corn steep liquor (CSL), casein, Mg^2+^ and Mn^2+^ ions, which resulted in protease activity of 188,956.23 U (698.24 mg protein) from an initial activity of 85,873.38 U (284.93 mg protein) by mutant kGy-04-UV-25. This indicates that the 2-LFD factorial design enhanced protease activity by 2.2-fold. The path of steepest ascent (PSA) increased protease activity to 306,945.52 U (1221.78 mg total protein). This indicated 1.62-fold increase from 2-LFD, 3.57-fold from mutation and 8.48-fold activity improvement from parent bacterium.

**Fig. 1:**
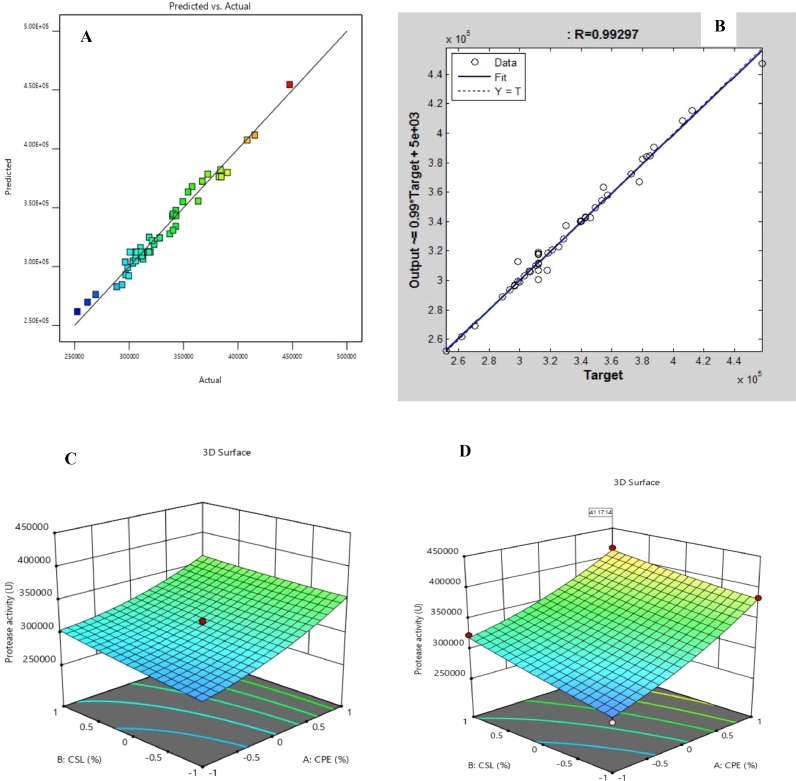
RSM (A) and ANN (B) model plots of actual versus predicted values and two-way RSM model (C) and optimized (D) interactions towards improved protease activity by mutant kGy-04-UV-25.

The design matrix, including actual values, experimental and predicted amounts of total protein (Y_1_) and protease activity (Y_2_) by RSM and ANN are presented in Table 2. Highest protease activity of 447,379 U (2413.46 mg protein) was observed in run 22 and was predicted as 456,000 U and (2643.58 mg protein) and 457,844.1 U (2702.05 mg prtotein) by RSM and ANN models respectively. We employed equations 5–11 for error metrics (Table 3) to compare the two models and results showed that apart from average absolute deviation (AAD) metric, ANN modeling outperformed RSM in prediction accuracy. The coefficient of determination, *r*^2^ of RSM model was 0.9749; *p* < 0.0001 suggesting that the model could account for 97.49% of the variability in the data about the experimental region (Fig. 1a). The lack of fit *F*-statistic, *F* (22, 29) = 1.94, *p* = 0.1876 was not significant at 5% significance level suggesting adequacy of the model to predict yield of protease by the mutant (Supplementary material, Table S1). The ANN model, developed by Levenberg-Marquardt back-propagation algorithm, with 12 neurons in its hidden layer (Supplementary material, Figure S1), had an *r*^2^ of 0.9859 indicating that only 1.42% of variations about the data would not be explained by the model (Fig. 1b). This, coupled with a comparatively low error metrics made ANN predictions more reliable. Previous studies have reported such superior prediction capability of ANN over RSM [14, 32]. The quadratic model for protease activity by the mutant is presented as Eq. (4) below.

(4) $$\begin{array}{c} Y_{2}=312416+34139.02X_{1}+8494.02X_{2}+3617.09X_{3}+6827.96X_{4}+3105.64X_{5}-4928.91X_{1}X_{2}-1433.03X_{1}X_{3}\\ +12458.59X_{1}X_{4}+6173.47X_{1}X_{5}+437.72X_{2}X_{3}+4314.59X_{2}X_{4}+6393.34X_{2}X_{5}-2139.91X_{3}X_{4}+1581.59X_{3}X_{5}\\ +442.72X_{4}X_{5}+10786.68{X_{1}}^{2}+1898.43{X_{2}}^{2}+178.84{X_{3}}^{2}+6132.32{X_{4}}^{2}+817.53{X_{5}}^{2} \end{array}$$

where Y_2_ is protease activity and X_1_ to X_5_ the significant variables of the model.

**Table 2:** Design matrix and responses of CCRD-RSM-ANN modeling of total protein and protease activity by strain kGy-04-UV-5.

Run	X1	X2	X3	X4	X5	Exp. Y_1_	RSM-Y_1_	ANN-Y_1_	Exp. Y_2_	RSM-Y_2_	ANN-Y_2_
1	45.7	23.2	6.93	0.38	0.039	935.12	1044.72	1170.4	261,830	268,400	262,288.8
2	46.9	23.9	6.13	0.27	0.031	1318.59	1253.49	1387.45	317,402	315,400	312,146.9
3	48.1	23.2	5.33	0.16	0.039	1693.47	1702.72	1616	340,385	346,600	340,269.2
4	46.9	23.9	6.13	0.27	0.031	998.22	1253.49	1387.45	300,848	315,400	312,146.9
5	46.9	23.9	6.13	0.05	0.031	1048.24	1331.74	1188.55	340,385	332,200	339,497
6	45.7	23.2	5.33	0.16	0.039	1351.35	1063.99	1064.1	269,275	281,100	270,420.3
7	48.1	24.6	6.93	0.38	0.039	2274.57	2196.8	2197.6	415,519	412,400	412,461.5
8	45.7	23.2	5.33	0.38	0.039	837.47	964.7	1066.6	252,400	261,200	251,729.8
9	46.9	23.9	6.13	0.27	0.047	1563.29	1619.47	1576.05	328,151	322,800	328,337.1
10	45.7	24.6	6.93	0.16	0.023	1219.06	1139.99	1155.05	310,405	312,700	310,336.8
11	45.7	24.6	5.33	0.38	0.039	1227.46	1287.64	1212.35	311,937	309,400	311,970.5
12	48.1	23.2	6.93	0.38	0.023	1948.38	1948.43	1893.25	367,211	378,000	377,815.2
13	45.7	23.2	6.93	0.16	0.039	1266.35	1211.33	1167.9	296,958	288,300	296,811.7
14	46.9	23.9	6.13	0.27	0.031	1355.33	1253.49	1387.45	319,375	315,400	312,146.9
15	46.9	23.9	6.13	0.27	0.031	1328.21	1253.49	1387.45	318,301	315,400	312,146.9
16	48.1	24.6	5.33	0.38	0.023	1893.29	1978.58	1935.15	372,208	373,800	372,361
17	46.9	23.9	7.73	0.27	0.031	1436.47	1490.05	1510.9	320,929	324,000	321,177.7
18	46.9	23.9	6.13	0.27	0.031	1187.41	1253.49	1387.45	306,867	315,400	312,146.9
19	46.9	23.9	4.53	0.27	0.031	1121.99	1243.1	1263.95	306,150	306,800	306,863.6
20	48.1	24.6	6.93	0.38	0.023	1983.25	2038.91	2039	384,720	381,000	384,641
21	46.9	23.9	6.13	0.27	0.031	1192.47	1253.49	1387.45	306,992	315,400	312,146.9
22	49.3	23.9	6.13	0.27	0.031	2413.46	2643.58	2702.05	447,379	456,000	457,844.1
23	44.5	23.9	6.13	0.27	0.031	994.46	939.02	997.5	299,714	293,600	298,634.8
24	45.7	24.6	5.33	0.16	0.023	973.46	936.68	1051.25	296,385	305,400	296,495.7
25	45.7	24.6	6.93	0.38	0.039	1522.12	1424.82	1316.2	323,043	316,700	325,167.7
26	45.7	24.6	5.33	0.16	0.039	1289.64	1265.05	1209.85	312,877	312,100	298,499.7
27	46.9	22.5	6.13	0.27	0.031	1076.47	1311.77	1214.1	303,431	295,200	303,242.4
28	48.1	23.2	5.33	0.38	0.039	2018.53	1932.93	1948	390,353	376,600	387,310.8
29	48.1	24.6	5.33	0.16	0.039	1819.38	1783.19	1761.8	349,332	357,900	349,119.9
30	46.9	23.9	6.13	0.49	0.031	1838.35	1729.53	1586.35	354,050	364,700	353,659.7
31	48.1	23.2	5.33	0.38	0.023	2093.4	1945.25	1789.4	382,518	370,800	379,807.3
32	45.7	23.2	5.33	0.38	0.023	1064.18	954.37	907.95	288,727	280,100	288,726.9
33	48.1	23.2	6.93	0.16	0.039	1978.46	1774.4	1719.85	363,476	353,900	354,450.7
34	45.7	24.6	5.33	0.38	0.023	1078.29	1108.29	1053.75	303,449	302,800	303,203.2
35	48.1	24.6	6.93	0.16	0.039	1884.38	1912.01	1865.6	357,922	365,200	357,339
36	48.1	24.6	5.33	0.16	0.023	1653.57	1477.48	1603.15	337,360	326,600	329,860.2
37	45.7	23.2	5.33	0.16	0.023	780.93	904.66	905.45	298,760	300,000	299,603.1
38	46.9	23.9	6.13	0.27	0.031	1320.48	1253.49	1387.45	318,272	315,400	312,146.9
39	48.1	23.2	5.33	0.16	0.023	1658.48	1566.04	1457.4	339,840	340,900	339,455.5
40	48.1	23.2	6.93	0.38	0.039	1937.27	1937.29	2051.85	383,862	383,800	382,572.9
41	46.9	23.9	6.13	0.27	0.031	1212.47	1253.49	1387.45	311,419	315,400	312,146.9
42	45.7	23.2	6.93	0.16	0.023	1129.31	1050.81	1009.3	306,356	307,200	306,191.7
43	46.9	23.9	6.13	0.27	0.015	1123.73	1242.23	1198.8	306,154	308,100	306,589
44	48.1	23.2	6.93	0.16	0.023	1708.39	1636.53	1561.25	342,792	348,100	342,818.5
45	45.7	23.2	6.93	0.38	0.023	1116.28	1033.21	1011.8	293,529	287,300	293,509.2
46	46.9	25.3	6.13	0.27	0.031	1719.08	1658.46	1560.8	343,103	335,600	343,017.9
47	48.1	24.6	6.93	0.16	0.023	1711.46	1605.11	1707	342,807	333,800	346,111.9
48	45.7	24.6	6.93	0.38	0.023	1125.08	1244.28	1157.55	306,881	310,000	317,813.1
49	48.1	24.6	5.33	0.38	0.039	2148.36	2135.29	2093.8	408,482	405,100	406,157.9
50	45.7	24.6	6.93	0.16	0.039	1342.46	1469.53	1313.7	318,496	319,300	318,389.3

X1, X2, X3, X4, X5 = Actual variables corresponding to cassava processing effluent (%), corn steep liquor (%), casein (%), magnesium ions (g/L) and manganese ions (g/L) respectively; Exp. – Experimental values obtained from CCRD experiments; RSM – Response surface methodology predicted values; ANN – Artificial neural network predicted values; Y1 = Total protein (mg); Y2 = Protease activity (U).

**Table 3:** Comparative performance of RSM and ANN models for improved protease yield by strain kGy-04-UV-25.

		Total protein		Protease activity	
S/N	Metric function	RSM	ANN	RSM	ANN
1	$R^{2}=1-\frac{\sum_{i=1}^{n}{\left(y-\hat{y}\right)}^{2}}{\sum_{i=1}^{n}{\left(y-\hat{y}\right)}^{2}}$ (5)	0.9154	0.9701	0.9749	0.986
2	$MSE=\frac{1}{n}\;\sum_{i=1}^{n}{\left(y-\hat{y}\right)}^{2}$ (6)	13,882.33	5187.03	48,520,112.31	21,571,562.43
3	$RMSE=\sqrt{\frac{1}{n}}\;\sum_{i=1}^{n}{\left(y-\hat{y}\right)}^{2}$ (7)	117.82	72.02	6965.64	4644.52
4	$MAE=\frac{1}{n}\sum_{i=1}^{n}\left|\hat{y}-y\right|$ (8)	95.86	38.78	5833.91	2755.26
5	$AAD=|\frac{1}{n}\sum_{i=1}^{n}\frac{y-y^{\prime }}{y^{\prime }}|*100$ (9)	0.06	0.16	0.01	0.07
6	$ARE=\frac{100}{n}\sum_{i=1}^{n}\frac{|y-y^{\prime }|}{y^{\prime }}$ (10)	7.15	3.22	1.79	0.825
7	$\chi 2=\sum_{i-1}^{n}{\left(\frac{y-y^{\prime }}{y^{\prime }}\right)}^{2}$ (11)	516.34	185.57	7395.77	3238.45

R2 – Coefficient of determination; MSE – Mean squared error; RMSE – Root mean squared error; MAE – Mean absolute error; AAD – Average absolute deviation; ARE – Average relative error; Pearson's Chi square error; *n* = number of samples; *y* = actual values; y’ = predicted values.

The optimization experiment gave a final protease activity of 411,713.87 U (2196.80 mg protein) by setting all factor levels as high (+1) which was validated in a triplicate shake flask experiment as 418,763.45 (2269.67 mg protein). Since the difference between protease yield by the response optimizer and the validated experiment was not up to 5% (only 1.71% difference), the optimum factor settings were accepted as true and employed to formulate medium for further investigations of protease production by *Stenotophomonas acidaminiphila* strain kGy-04-UV-25. Fermentation medium for protease production by the study mutant in the 5-L bioreactor was therefore composed of 48.1% treated-CPE, 26.4% CSL, 6.93% casein, 0.38 gL^−1^ Mg^2+^ and 0.039 gL^−1^ Mn^2+^ as significant nutrients. Selection of two agro-based substrates namely CPE and CSL, in fermentation medium confers some techno-economic benefits on the process and doubles as a sustainable means of combating environmental pollution due to these wastes. Successful production of proteases on agro-industrial wastes to improve production economics has been reported by Hammami et al. [13].

### 3.3 Kinetics of protease production by strain kGy-04-UV-25 in a 5-L bioreactor

**Fig. 2:**
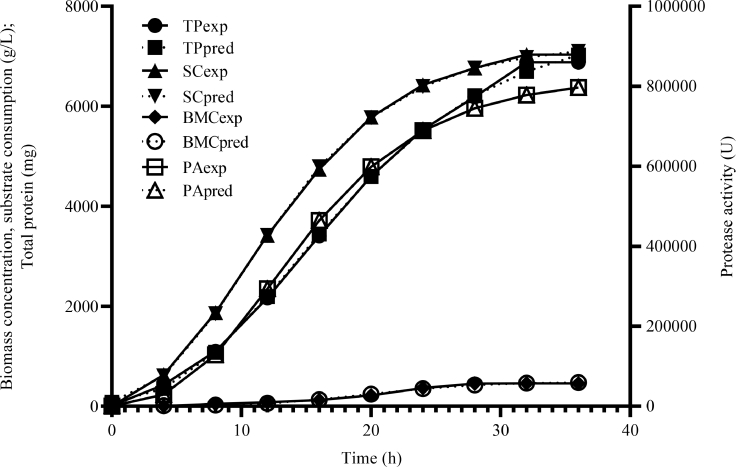
Logistic and modified Gompertz model-fitting of biomass concentration (BMC), total protein (TP), protease activity (PA) and substrate consumption (SC) data. Exp. – Experimental data; Pred. – Predicted data.

Details of the kinetics of fermentation are presented in Fig. 2 and Table 4. Several models including Luderking-Piret, Monod and modified Gompertz models were employed to fit BMC data, alongside logistic model. In the end, BMC data was best fitted by the logistic model while protease formation, protease activity and substrate consumption were best fitted by modified Gompertz model. Fig. 2 shows that exponential biomass formation did not commence until about 5 h. The figure also shows that for the most part of fermentation, BMC continued to increase suggesting that a good amount of substrate consumed by the bacterium was channeled into cellular metabolism until 32 h when the population entered stationary growth phase with a maximum BMC of 455 g/L. Table 4 shows that the mutant grew at a maximum specific growth rate, *µ*_max_ of 0.578 *h*^−1^. Comparative specific growth rates of mutant in Luria-Bertani (0.576 *h*^−1^) and trypticase soy (0.591 *h*^−1^) broths were not statistically different from those obtained in the optimized medium. However, they were significantly (*p* < 0.05) faster than those of parental strain in all three media: fermentation medium (0.362 *h*^−1^), Luria-Bertani (0.358 *h*^−1^) and trypticase soy (0.356 *h*^−1^). This confirms that growth rate is both a function of medium and genetic capability of the bacterium. Biomass yield relative to amount of substrate consumed, Yx/s was calculated as 0.067 gg^−1^. The logistic model has been employed by previous researchers to fit bacterial growth data [18].

**Table 4:** Bioreactor kinetic model parameters and statistics summary of fermentation response data for wild type and mutant strains of *Stenotrophomonas acidaminiphila* strain UCCM 00,065 in optimized medium.

Model	Biomass formation model parameters								Performance statistics of model			
LGM	*X*_0_	*X*_max_	µ_max_	g					Adj. r^2^	RMSE	MAE	*p*-value
Mutant	3.29 ± 0.28	482.46 ± 27.36	0.578 ± 0.01	0.36 ± 0.01					0.9909	17.00	13.90	<0.0001
Wild type	0.61 ± 0.03	228.65 ± 18.46	0.362 ± 0.01	0.522 ± 0.01					0.9867	10.66	7.79	<0.0001
	Protease formation and activity model parameters.l								Performance statistics of mode			
MGM	*P*_max_	rp_max_	t_L_						Adj. r^2^	RMSE	MAE	*p*-value
Mutant	7644.62 ± 108.32	315.46 ± 32.43	5.07 ± 0.64	Total protein formation					0.9989	81.04	56.89	<0.0001
Wild type	132.05 ± 5.32	27.55 ± 2.84	7.15 ± 0.86						0.9703	8.84	7.11	<0.0001
Mutant	820,953.05 ± 26,388.52	44,529.23 ± 3527.11	5.41 ± 0.32	Protease activity					0.999	624.90	242.49	<0.0001
Wild type	76.73 ± 11.83	28.16 ± 9.82	7.07.36 ± 1.04						0.984	7.25	6.78	<0.0001
	Substrate consumption model parameters									Performance statistics of model		
MGM	*S*_max_	rs_max_	t_L_	Yp/s	Yx/s	Yp/x	q_p_	q_s_	Adj. r^2^	RMSE	MAE	*p*-value
Mutant	7250.25 ± 122.67	397.64 ± 30.23	3.38 ± 0.37	1.054 ± 0.03	0.067 ± 0.00	0.016 ± 0.00	0.511 ± 0.04	0.461 ± 0.06	0.9997	43.17	34.16	<0.0001
Wild type	2919.22 ± 32.17	202.20 ± 12.53	2.51 ± 0.25	0.045 ± 0.00	0.078 ± 0.00	0.00058 ± 0.00	0.02 ± 0.00	0.381 ± 0.01	0.9770	150.45	109.31	<0.0001

LGM = Logistic model; MGM = Modified Gompertz model; P_max_ = maximum protease concentration (mg) or activity (U); r_pmax_ = maximum volumetric rate of protease formation (mgL^−1^h^−1^) or activity (UL^−1^h^−1^); X0 = initial biomass concentration (gL^−1^); Xmax = maximum biomass concentration (gL^−1^); µmax = maximum specific growth rate (*h*^−1^); tLag = Lag time (h); Smax = Maximum substrate consumption (gL^−1^); rsmax = maximum volumetric rate of substrate consumption (gL^−1^h^−1^); Adj. r2 = adjusted coefficient of determination; RMSE = root mean squared error; MAE = mean absolute error; Yp/*s* = Protease yield relative to amount of substrate consumed (mgg−1); Yx/*s* = Biomass yield relative to amount of substrate consumed (gg^−1^); Yp/*x* = Specific protease yield (gg^−1^); qp = specific rate of protease formation (mggDCW^−1^h^−1^); qs = Specific rate of substrate consumption (ggDCW^−1^h^−1^). The kinetic paraeter values are means of triplicate determinations ± standard error.

Lag time, t_L_, for protease accumulation was 5 h, just about the time biomass entered exponential phase of growth. This led to a final protease yield of 6879.67 mg by the 32^nd^ hour (Fig. 2) with a corresponding activity of 796,890 U. The lag time is defined as the time taken for the rate of growth/production of the bacterium/metabolite to accelerate beyond the minimum asymptote where growth/synthesis started and attain maximum specific growth/production rate [33]. Maximum achievable protease activity was 820,953.05 U from 7644.62 mg protein compared to 36,209.54 U from 132.05 mg protein in the parent. This implies that protease activity in the mutant improved 22-fold. The modified Gompertz model for protease activity was significant with an *r*^2^ of 0.999, *p* < 0.0001 and a mean absolute error (MAE) of 242.49. Only very few researches report on protease concentrations this high after a series of optimizations. Secades and Guijarro [34] reported a total protein of 4400 mg from *Yersinia ruckeri*, with total protease activity of 412,533 U. A bivariate correlation analysis of the four response variables revealed significant (*p* < 0.0001) multi-colinearity (*r* = 0.99) between each pair of responses suggesting that the conditions employed to achieve the amount of total protein would very well suffice for protease activity.

Formation of biomass (BMC) and product (protease) are a function of substrate consumption. The modified Gompertz model revealed that 7250.25 gL^−1^ of reducing sugar could be consumed by the mutant over 36 h fermentation at a volumetric consumption rate of 397.64 gL^−1^h^−1^ compared to 202.20 gL^−1^h^−1^ by wild type. These results indicate that maximum consumable reducing sugar in the mutant increased 2.48-fold from that of the parent. The model was significant with an *r*^2^ of 0.9997 and MAE of 34.16. Table 4 shows that specific rate of substrate consumption of 0.461 ggDCW^−1^h^−1^ was in reasonable agreement with specific rate of protease formation (0.511 mggDCW^−1^h^−1^) and that for every gram of reducing sugar consumed by the mutant a milligram of protease would be synthesized. Specific yields of biomass and product on substrate were encouraging and firmly support the feasibility of scaling the bioprocess to the pilot level.

### 3.4 Partial-purification of protease by aqueous two-phase system

The application of aqueous two-phase system in the purification of proteins, especially enzymes, was documented by Iqbal et al. [35]. Table 5 shows that 20% (ww^−1^) of 1500 mol.wt. polyethylene glycol (PEG) combined with 15% Na^+^ citrate at 55°C and pH 10.5, recovered protease with most activity yield of 73.87% and purity of 52.55. This agrees with the works of Wongmongkol and Prichanont [36] who reported protease extraction with lower molecular weight PEG-1000 with commendable phase separation. Highest enzyme activity, following protein precipitation, occurred in the top phase as observed by Chouyyok et al. [37] who extracted *Bacillus subtilis* strain TISTR 25 alkaline protease using the method. Interestingly, there was only slight but significant difference (*p* > 0.05) between PEG 3000 and 6000 in terms of yield, although purification folds were significantly different (*p* < 0.001) and no clear pattern could be found. Optimization of PEG molecular weight, salt concentration, temperature and pH is recommended for selection of optimal conditions towards achieving a higher yield/purity balance.

**Table 5:** Effect of molecular weight (mol.wt.) of polyethylene glycol (PEG) on specific activity, yield and fold of protease from aqueous two-phase system (ATPS) purification.

Purification step	PEG Mol.wt	Total protein (mg)	Total activity (U)	Specific activity (U/mg)	Yield (%)	Fold purification
Sterile crude extract ATPS with PEG/Na^+^Citrate (20% PEG/15% Na^+^ citrate/55°C/pH 10.5)	0	6879.45	796,890	115.84	100.00	1.00
	1500	96.71	588,673.75	6087.00	73.87	52.55
	3000	243.97	493,823.36	2024.12	61.97	17.43
	4500	312.73	328,834.03	1051.50	41.27	9.08
	6000	152.19	506,459.74	3327.81	63.56	28.73
	7500	453.96	234,979.12	517.61	29.49	4.47

PEG – Polyethylene glycol; Mol.wt – Molecular weight; ATPS – Aqueous two-phase system.

### 3.5 SDS-PAGE determination of molecular weight of protease

**Fig. 3:**
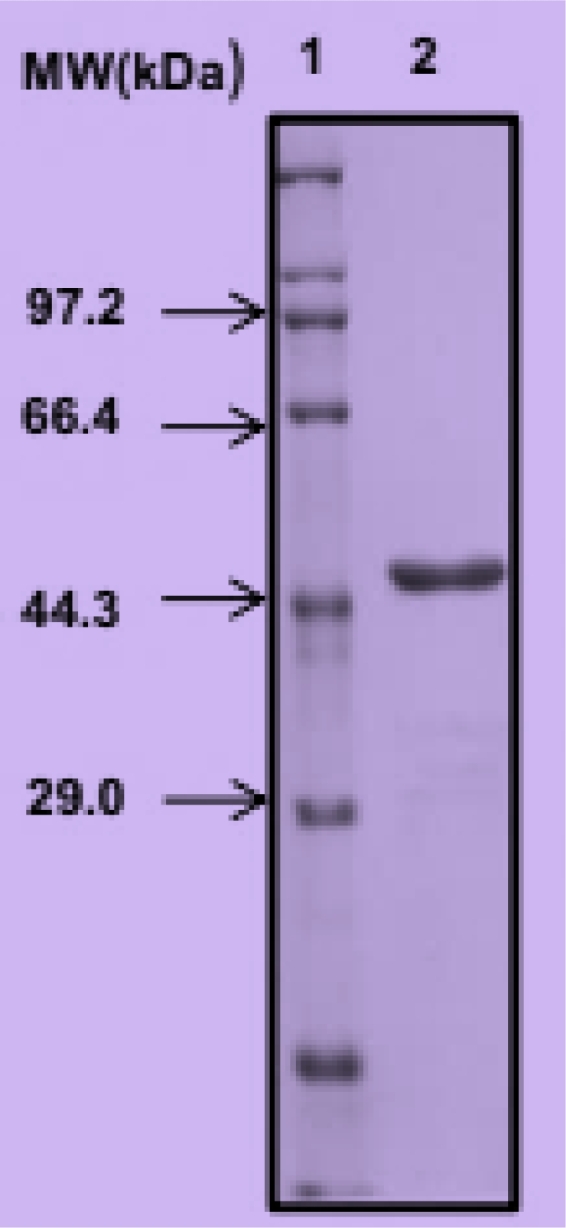
SDS-PAGE molecular weight (protein standards in lane 1) determination of Stenotrophomonas acidaminiphila strain kGy-04-UV-25 alkaline protease (lane 2).

Results of the SDS-PAGE determination of molecular weight of the study protease revealed a single band corresponding to 45.7 kDa as presented in Fig. 3. In lane 1 are standard proteins with known molecular weights while lane 2 shows the single band of the purified study protein occurring just above protein with 44.3 kDa. The 45.7 kDa protease was significantly smaller than the 98 kDa from *Stenotrophomonas maltophila* strain SK [8] but not that different from the 45 kDa of *Stenotrophomonas maltophila* of Wang et al. [38]. This report could serve as base for further studies on protease production by *Stenotrophomonas acidaminiphila*. The single band on SDS-PAGE suggests that although ATPS is a first-step purification process, careful selection of conditions could purify the protein sufficiently for determination of important characteristics.

### 3.6 Physico-chemical characteristics of mutant kGy-04-UV-25 protease

**Fig. 4:**
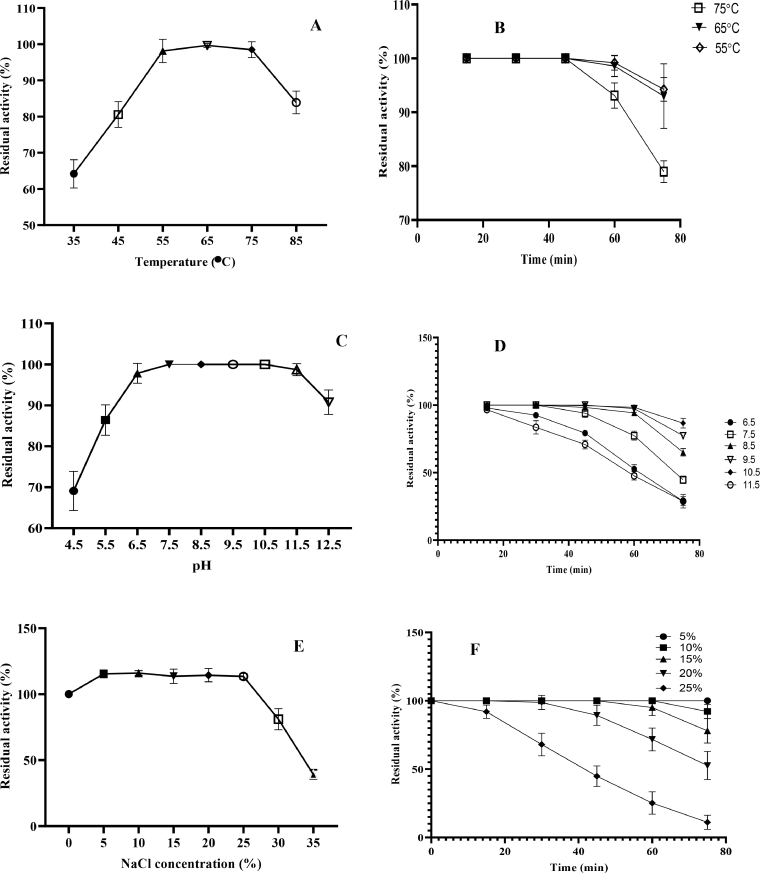
Physicochemical properties of Stenotrophomonas acidaminiphila mutant kGy-04-UV-5 protease.

Studies to determine temperature, pH and NaCl optima and their stabilities are presented in Fig. 4 (a-f). Fig. 4a shows that optimum temperature for protease activity ranged from 55 to 75°C making it thermo-stable; the stability of which lasted for 60 min at 55–65°C. At 75°C, 79% of the activity was retained after exposure for 75 min (Fig. 4b). The alkaline protease from *Bacillus* sp. strain B18 has been reported to have a temperature optimum of 85°C [39]. The study protease retained 83.9% of its activity at 85°C. Many *Bacillus* proteases have activity optima lower than 75°C giving the protease in this study significant advantage. Fig. 4c shows that optimum pH range for protease activity was 6.5–11.5; a very wide alkaline range, making the protease strongly alkaline [39]. Protease activity, after pre-incubation at pH 11.5, was not significantly different (*p* = 0.9988 > 0.05) from those at 6.5–10.5 but was significantly different (*p* = 0.0193 <  0.05) from activity at pH 12.5. Activity at different pH optima was stable for different lengths of exposure time (Fig. 4d). Protease activities after pre-incubation at pH 9.5 and 10.5 were stable for 60 min while those from pH 6.5 and 11.5 retained respectively, 92 and 83% activities after 30 min exposure. For the duration of industrial application, particularly laundry, the thermo-stable alkaline protease reported here will retain most of its activity. Optimum NaCl concentration for protease activity was found in the range 5–35% suggesting that the protease was stable to saline conditions (Fig. 4e). Activity actually increased after exposure to 5% NaCl and was stable until 30% NaCl. However, halo-stability (Fig. 4f) was gradually lost at 25% NaCl with 91.98% of activity retained after 15 min, 68.04% at 30 min and 25.22% at 45 min.

### 3.7 Stability of protease to inhibitors, divalent cations, organic solvents and denaturants

**Fig. 5:**
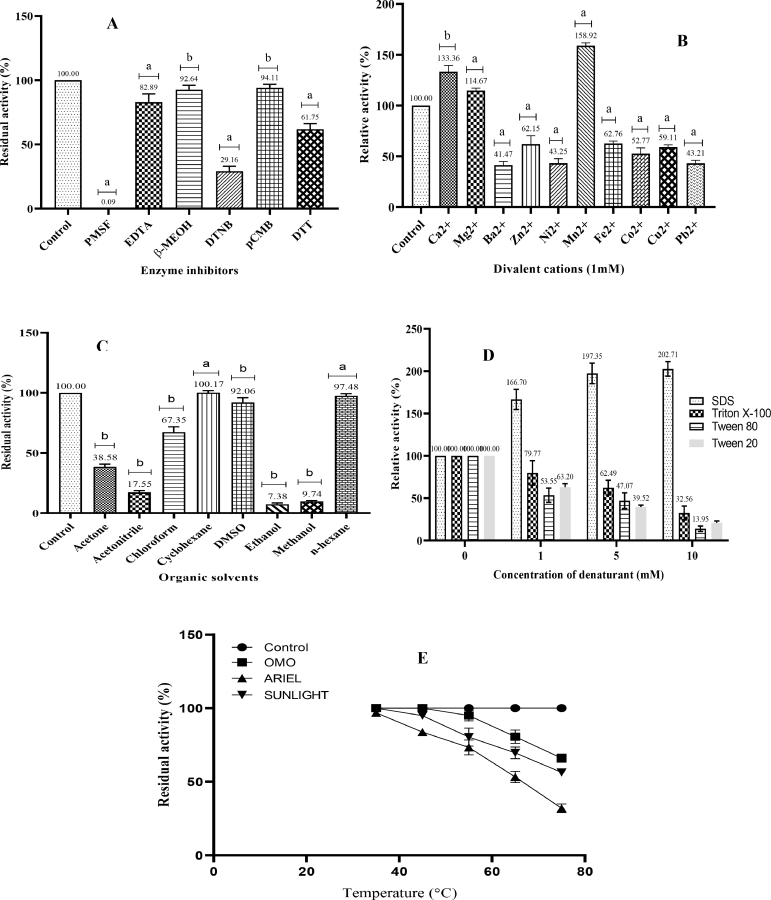
Stability of thermo-stable halo-alkaline protease to (A) inhibitors (B) divalent cations (C) organic solvents (D) denaturants and (E) commercial detergents PMSF – Phenylmethylsulfonyl fluoride; EDTA – Ethylene diamine tetraacetic acid; β-MEOH – β-mercaptoethanol; DTNB – dithio-bis-nitrobenzoic acid; pCMB – p‑chloro mercuric benzoate; DTT – dithiothreitol; a – significant; b – not significant at *p* = 0.05; Plotted values are means of triplicate determinations; error bars are standard deviations.

Fig. 5a reveals the susceptibility of the study thermo-stable halo-alkaline protease to inhibitors. The most potent inhibitor at 5 mM concentration was PMSF (the well-known serine‑protease inhibitor), followed by DNTB both of which produced residual activities of 0.09% and 29.16% respectively. Öztürk et al. [40] reported complete loss of activity when their alkaline protease from *Bacillus* sp. strain BA17 was exposed to 1–5 mM of PMSF as observed in this study. The inhibitor, PMSF is known to sulfonate serine residues at active sites of the enzyme bringing about complete loss of activity [38]. The inhibition by PMSF, coupled with the low molecular weight of 45.7 kDa, go to suggest the protease as a serine‑protease. β-mercaptoethanol and *p*‑chloro-mercuric benzoate did not significantly inhibit protease activity at 5 mM. This report also agrees with that of Guleria et al. [41] who reported similar ranges of inhibition of the alkaline protease from *Bacillus amyloliquefaciens* strain SP1.

Results of the effects of divalent cations on protease activity are presented in Fig. 5b. The figure shows that Ca^2+^, Mg^2+^ and Mn^2+^ enhanced activity of protease at 1 mM concentration, with Mn^2+^showing the most enhancement of 58.92%, followed by 33.6% and 14.67% for Ca^2+^ and Mg^2+^ respectively. All other metal ions significantly inhibited the enzyme. This suggests requirement for Ca^2+^, Mn^2+^ and Mg^2+^ at the active site of the study protease for protection against denaturation from heat or denaturants and/or cofactor requirements of the enzyme. Inhibition of protease activity was observed with Zn^2+^, Ni^2+^, Pb^2+^ and Ba^2+^ [24].

Activity of mutant kGy-04-UV-25 protease was tested against six organic solvents and results, presented in Fig. 5c, reveal that ethanol, methanol and acetonitrile were the most inhibitory organic solvents since only 7.38, 9.74 and 17.55% activities were respectively retained after 24 h of pre-incubation with these solvents. Cyclohexane and n-hexane did not significantly inhibit enzyme activity. It appears that solvents like ethanol, methanol, acetonitrile and acetone with low log *P* values of -0.24, -0.76, -0.15 and -0.23 respectively could very easily rid the enzyme of its activity. n-hexane and cyclohexane with log *P* values close to 4.0 did not demonstrate any significant inhibition of protease activity even after 72 h of incubation. These results are in consonance with those reported by Mhamdi et al. [42].

The results of residual activities of the enzyme after exposure to denaturants aimed at demonstrating suitability of protease for laundry applications are presented as Fig. 5d. The figure reveals that Triton X-100, Tween 20 and Tween 80 denatured the protease at all concentrations resulting in loss of activity relative to the control. However, SDS enhanced the activity of the protease with increasing surfactant concentration. Pre-incubation of the enzyme with 10 mM SDS resulted in 92.7% significant enhancement of activity relative to the control (100%). These results are in concord with a number of findings for other bacteria [37, 41].

The compatibility evaluation of study protease with commercial detergents shows that OMO was the most compatible detergent as the protease was able to retain 80% of its activity at 65°C (Fig. 5e). These results agree with that of Mhamdi et al. [42] who showed that the alkaline protease of *Bacillus safensis* strain S406 could retain 95% of its activity showing strong compatibility with the detergent. However, the study protease did not demonstrate such compatibility with ARIEL which caused the loss of 26.7% of protease activity at that temperature.

### 3.8 Potential applications of protease

**Fig. 6:**
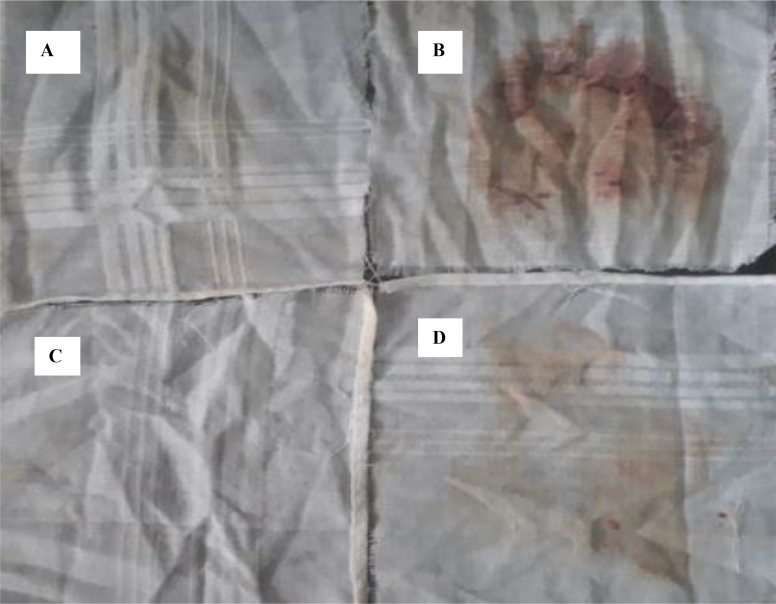
Blood stain removal potential of thermo-stable halo-alkaline protease by Stenotrophomonas acidaminiphila strain kGy-04-UV-25.A-Blood stained cloth washed with 5 mg/mL of protease alone; B – Blood stained cloth washed with 100 mL distilled water; C – Blood stained cloth washed with 5 mgmL−1 protease and 5 mgL−1 detergent (OMO) and D - Blood stained cloth washed with 5 mgL−1 detergent (OMO) alone.

Results of keratinolytic assay revealed that the thermo-stable halo-alkaline protease from the study mutant showed profound keratinolytic activity of 10,953 U with the crude culture filtrate. The purified product, however, demonstrated 74,056 U of activity. The blood stain removal potential of the protease is presented as Fig. 6 and shows clearly that the study protease could be used as an additive in detergent formulations. Similar findings have been reported by Annamalai et al. [2] with thermo-stable halo-alkaline, solvent-stable protease from *Bacillus halodurans* strain CAS6.

## 4 Conclusions

The mutant, *Stenotrophomonas acidaminiphila* strain kGy-04-UV-25, generated by alternate combinatorial random mutagenesis with gamma and ultraviolet rays produced a thermo-stable halo-alkaline protease on pretreated cassava processing effluent and corn steep liquor as carbon and nitrogen sources, respectively. Modeling and optimization of medium conditions using RSM and ANN significantly improved enzyme activity by several folds in shake flasks which was successfully scaled up in a 5-L bioreactor by 22-fold. The 45.7 kDa manganese-dependent thermo-stable halo-alkaline serine‑protease recovered by aqueous two-phase system had its activity enhanced by SDS but inhibited by PMSF. Its keratinolytic activity could be exploited in the textile industry and its blood stain removal potential, as an additive in detergent formulations.

## Funding

This research did not receive any specific grant from funding agencies in the public, commercial, or not-for-profit sectors.

## Declaration of Competing Interests

The authors declare that they have no known competing financial interests or personal relationships that could have appeared to influence the work reported in this paper. The authors declare that they have no known competing financial interests or personal relationships that could have appeared to influence the work reported in this paper.

## Appendix Supplementary materials

Image, application 1
