# Supplementary material for: Overproduction of a thermo-stable halo-alkaline protease on agro-waste-based optimized medium through alternate combinatorial random mutagenesis of Stenotrophomonas acidaminiphila
Source: Biotechnol Rep (Amst). 2022 Jun 5;35:e00746. doi: 10.1016/j.btre.2022.e00746 (PMC9189783; doi:10.1016/j.btre.2022.e00746)
Supplement: Supplementary file 1 [file mmc1.docx]

**SUPPLEMENTARY INFORMATION**

**Overproduction of a thermo-stable halo-alkaline protease on agro-waste-based optimized medium through alternate combinatorial random mutagenesis of *Stenotrophomonas acidaminiphila***

Atim Asitok^1,2^, Maurice Ekpenyong^1,2^*, Iquo Takon^3^, Sylvester Antai^1,2^, Nkpa Ogarekpe^4^, Richard Antigha^4^, Philomena Edet^1^, Ubong Ben^5^, Anthony Akpan^5^ Agnes Antai^6^ & Joseph Essien^7^

1. Environmental Microbiology and Biotechnology Unit, Department of Microbiology, Faculty of Biological Sciences, University of Calabar, Nigeria
2. University of Calabar Collection of Microorganisms (UCCM), Department of Microbiology, University of Calabar, Nigeria
3. Industrial Microbiology and Biotechnology Unit, Department of Microbiology, Faculty of Biological Sciences, University of Calabar, Nigeria
4. Environmental Engineering Unit, Department of Civil Engineering, Faculty of Engineering, Cross River University of Technology, Nigeria
5. Department of Physics, Faculty of Physical Sciences, University of Calabar, Nigeria
6. Department of Economics, Faculty of Social Sciences, University of Calabar, Nigeria
7. Environmental Microbiology and Biotechnology Unit, Department of Microbiology, Faculty of Sciences, University of Uyo, Nigeria

*Correspondence: *ME: [maurygg2002@yahoo.com](mailto:maurygg2002@yahoo.com), [mauriceekpenyong@unical.edu.ng](mailto:mauriceekpenyong@unical.edu.ng); +234-70-38218972; ORCID No.: <https://orcid.org/0000-0001-9601-5546>

The tables and figures were obtained from the study which caption appears at the top of this page. Response surface methodology was employed to model the results of a central composite rotatable design and Table S1 is the ANOVA table of the multiple regressions with lack-of-fit of the model. Table S2 presents the coefficients table from which predictor coefficients were obtained to build the regression model for protease activity. Figure 1a is the artificial neural network topology built using 12 neurons (2n + 2) in the hidden layer while the performance of the network is presented as Figure 1b. The regression values used to evaluate model performance and which establishes a correlation between actual and predicted values are given in Figure 2. It is to be emphasized that the *R* presented by the network is not the coefficient of determination which would be *R*^2^. Therefore, the overall *R* obtained in this figure as 0.99297 was squared to obtain the *R*^2^ (goodness-of-fit) of the ANN model for comparison with *R*^2^ from RSM model.

**Table S1 ANOVA for response surface modeling (RSM) of protease activity by mutant kGy-04-UV-25**

| **Source** | **Sum of Squares** | **df** | **Mean Square** | ***F*-value** | ***p*-value** |
| --- | --- | --- | --- | --- | --- |
| **Model** | 7.374E+10 | 20 | 3.687E+09 | 56.23 | < 0.0001 |
| A-CPE | 5.048E+10 | 1 | 5.048E+10 | 769.80 | < 0.0001 |
| B-CSL | 3.125E+09 | 1 | 3.125E+09 | 47.65 | < 0.0001 |
| C-Casein | 5.667E+08 | 1 | 5.667E+08 | 8.64 | 0.0064 |
| D-Mg^2+^ | 2.019E+09 | 1 | 2.019E+09 | 30.79 | < 0.0001 |
| E-Mn^2+^ | 4.178E+08 | 1 | 4.178E+08 | 6.37 | 0.0173 |
| AB | 7.774E+08 | 1 | 7.774E+08 | 11.85 | 0.0018 |
| AC | 6.571E+07 | 1 | 6.571E+07 | 1.00 | 0.3251 |
| AD | 4.967E+09 | 1 | 4.967E+09 | 75.74 | < 0.0001 |
| AE | 1.220E+09 | 1 | 1.220E+09 | 18.60 | 0.0002 |
| BC | 6.131E+06 | 1 | 6.131E+06 | 0.0935 | 0.7620 |
| BD | 5.957E+08 | 1 | 5.957E+08 | 9.08 | 0.0053 |
| BE | 1.308E+09 | 1 | 1.308E+09 | 19.95 | 0.0001 |
| CD | 1.465E+08 | 1 | 1.465E+08 | 2.23 | 0.1458 |
| CE | 8.005E+07 | 1 | 8.005E+07 | 1.22 | 0.2783 |
| DE | 6.272E+06 | 1 | 6.272E+06 | 0.0956 | 0.7593 |
| A² | 6.466E+09 | 1 | 6.466E+09 | 98.60 | < 0.0001 |
| B² | 2.003E+08 | 1 | 2.003E+08 | 3.05 | 0.0911 |
| C² | 1.777E+06 | 1 | 1.777E+06 | 0.0271 | 0.8704 |
| D² | 2.090E+09 | 1 | 2.090E+09 | 31.87 | < 0.0001 |
| E² | 3.714E+07 | 1 | 3.714E+07 | 0.5664 | 0.4578 |
| **Residual** | 1.902E+09 | 29 | 6.558E+07 |  |  |
| Lack of Fit | 1.564E+09 | 22 | 7.111E+07 | 1.48 | 0.3107 |
| Pure Error | 3.372E+08 | 7 | 4.818E+07 |  |  |
| **Cor Total** | 7.564E+10 | 49 |  |  |  |

CPE - Cassava processing effluent; CSL - Corn steep liquor

**Table S2 Coefficients of predictors of protease activity model used to build the model equation**

| **Factor** | **Coefficient Estimate** | **df** | **Standard Error** | **95% CI Low** | **95% CI High** | **VIF** |
| --- | --- | --- | --- | --- | --- | --- |
| Intercept | 3.124E+05 | 1 | 2841.25 | 3.066E+05 | 3.182E+05 |  |
| A-CPE | 34139.02 | 1 | 1230.45 | 31622.47 | 36655.57 | 1.0000 |
| B-CSL | 8494.02 | 1 | 1230.45 | 5977.47 | 11010.57 | 1.0000 |
| C-Casein | 3617.09 | 1 | 1230.45 | 1100.54 | 6133.64 | 1.0000 |
| D-Mg^2+^ | 6827.95 | 1 | 1230.45 | 4311.41 | 9344.50 | 1.0000 |
| E-Mn^2+^ | 3105.64 | 1 | 1230.45 | 589.10 | 5622.19 | 1.0000 |
| AB | -4928.91 | 1 | 1431.53 | -7856.72 | -2001.10 | 1.0000 |
| AC | -1433.03 | 1 | 1431.53 | -4360.84 | 1494.78 | 1.0000 |
| AD | 12458.59 | 1 | 1431.53 | 9530.78 | 15386.40 | 1.0000 |
| AE | 6173.47 | 1 | 1431.53 | 3245.66 | 9101.28 | 1.0000 |
| BC | 437.72 | 1 | 1431.53 | -2490.09 | 3365.53 | 1.0000 |
| BD | 4314.59 | 1 | 1431.53 | 1386.78 | 7242.40 | 1.0000 |
| BE | 6393.34 | 1 | 1431.53 | 3465.53 | 9321.15 | 1.0000 |
| CD | -2139.91 | 1 | 1431.53 | -5067.72 | 787.90 | 1.0000 |
| CE | 1581.59 | 1 | 1431.53 | -1346.22 | 4509.40 | 1.0000 |
| DE | 442.72 | 1 | 1431.53 | -2485.09 | 3370.53 | 1.0000 |
| A² | 10786.68 | 1 | 1086.33 | 8564.89 | 13008.46 | 1.05 |
| B² | 1898.43 | 1 | 1086.33 | -323.35 | 4120.22 | 1.05 |
| C² | 178.84 | 1 | 1086.33 | -2042.95 | 2400.62 | 1.05 |
| D² | 6132.32 | 1 | 1086.33 | 3910.54 | 8354.11 | 1.05 |
| E² | 817.53 | 1 | 1086.33 | -1404.25 | 3039.32 | 1.05 |

CPE - Cassava processing effluent; CSL - Corn steep liquor; df – Degrees of freedom; CI = confidence interval; VIF = Variance inflation factor


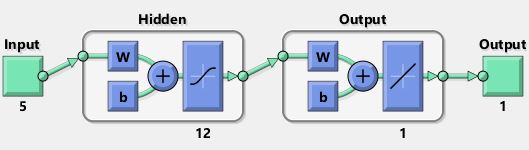


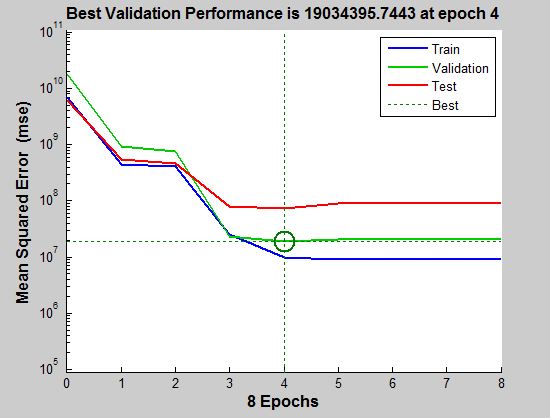


Fig. 1 Artificial neural network (ANN) topology (A) and performance (B) for modeling protease activity of mutant kGy-04-UV-25


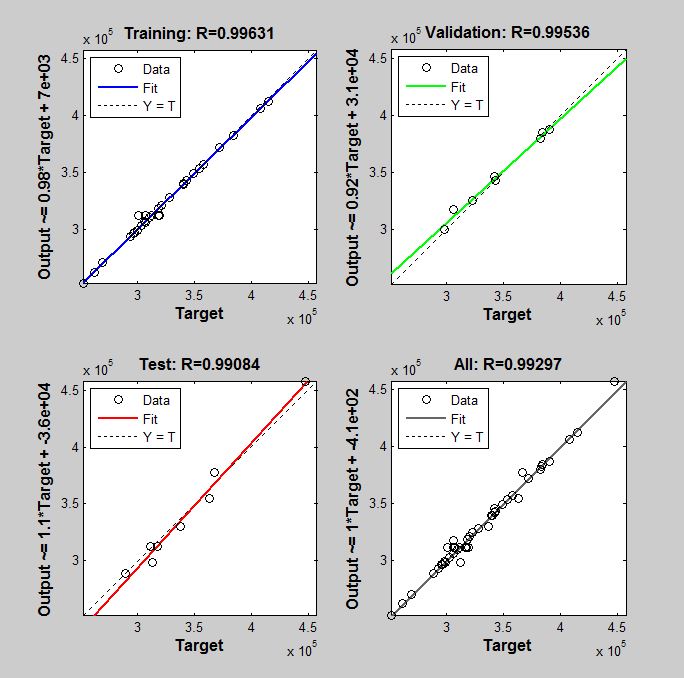


Fig. 2 Regression results for training, validation and testing of neural networks with their summary indicated by ALL

**References**

Kanno, A.I., Leite, L.C.C., Pereira, L.R., Rodrigues de Jesus, M.J., Andreata‑Santos, R., Alves, R.P.D.S., Durigon, E.L., Ferreira, L.C.S., Gonçalves, V.M., 2020. Optimization and scale‑up production of Zika virus ΔNS1 in *Escherichia coli*: application of response surface methodology. AMB Expr 10: 1.

Ekpenyong, M.G., Asitok, A.D., Antai, S.P., Ekpo, B.O., Antigha, R.E., Ogarekpe, N.M., 2021. Statistical and artificial neural network approaches to modeling and optimization of fermentation conditions for production of a surface/bioactive glyco-lipo-peptide. Int. J. Pept. Res. Ther. 27, 475-495

Karri, R.R., Sahu, J.N., 2018. Modeling and optimization by particle swarm embedded neural network for adsorption of zinc (II) by palm kernel shell based activated carbon from aqueous environment. J. Environ. Manage. 206, 178-191.
